# Mixed Acid-Base Disorders, Hydroelectrolyte Imbalance and Lactate Production in Hypercapnic Respiratory Failure: The Role of Noninvasive Ventilation

**DOI:** 10.1371/journal.pone.0035245

**Published:** 2012-04-23

**Authors:** Claudio Terzano, Fabio Di Stefano, Vittoria Conti, Marta Di Nicola, Gregorino Paone, Angelo Petroianni, Alberto Ricci

**Affiliations:** 1 Fondazione Eleonora Lorillard Spencer Cenci, Sapienza University of Rome, Rome, Italy; 2 Laboratory of Biostatistics, Department of Biomedical Science, University “G. d'Annunzio” of Chieti-Pescara, Chieti, Italy; University of Pittsburgh, United States of America

## Abstract

**Background:**

Hypercapnic Chronic Obstructive Pulmonary Disease (COPD) exacerbation in patients with comorbidities and multidrug therapy is complicated by mixed acid-base, hydro-electrolyte and lactate disorders. Aim of this study was to determine the relationships of these disorders with the requirement for and duration of noninvasive ventilation (NIV) when treating hypercapnic respiratory failure.

**Methods:**

Sixty-seven consecutive patients who were hospitalized for hypercapnic COPD exacerbation had their clinical condition, respiratory function, blood chemistry, arterial blood gases, blood lactate and volemic state assessed. Heart and respiratory rates, pH, PaO_2_ and PaCO_2_ and blood lactate were checked at the 1st, 2nd, 6th and 24th hours after starting NIV.

**Results:**

Nine patients were transferred to the intensive care unit. NIV was performed in 11/17 (64.7%) mixed respiratory acidosis–metabolic alkalosis, 10/36 (27.8%) respiratory acidosis and 3/5 (60%) mixed respiratory-metabolic acidosis patients (p = 0.026), with durations of 45.1±9.8, 36.2±8.9 and 53.3±4.1 hours, respectively (p = 0.016). The duration of ventilation was associated with higher blood lactate (p<0.001), lower pH (p = 0.016), lower serum sodium (p = 0.014) and lower chloride (p = 0.038). Hyponatremia without hypervolemic hypochloremia occurred in 11 respiratory acidosis patients. Hypovolemic hyponatremia with hypochloremia and hypokalemia occurred in 10 mixed respiratory acidosis–metabolic alkalosis patients, and euvolemic hypochloremia occurred in the other 7 patients with this mixed acid-base disorder.

**Conclusions:**

Mixed acid-base and lactate disorders during hypercapnic COPD exacerbations predict the need for and longer duration of NIV. The combination of mixed acid-base disorders and hydro-electrolyte disturbances should be further investigated.

## Introduction

Hypercapnic respiratory failure is a complex condition associated with the malfunction of various organs and systems crucial for many physiological processes, leading to an acid-base imbalance.

Carbon dioxide (CO_2_) is not the only independent variable that may cause alterations in acid-base status. Total serum protein, albumin in particular, plays an important role, as does the strong ion difference (SID), that is the difference between the strong positive ions in the plasma (sodium (Na^+^), potassium (K^+^), calcium (Ca^2+^), and magnesium (Mg^2+^)) and the strong negative ions (chloride (Cl^−^) and lactate (Lac^−^)):
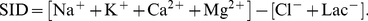
(1)At pH 7.4, 37°C and a partial carbon dioxide tension of 40 mmHg, the ideal value of SID is 42 mmol/L . An increased SID causes alkalosis; a reduced SID causes acidosis. Altering SID means altering the water dissociation equilibrium. This provides more/less H^+^ for electroneutrality, with a change in [H^+^], and so a change in pH.

Acid-base and electrolyte balance are part of the same picture because, for a given increase in CO_2_, the only way to minimize the resulting acidemia is to produce compensatory metabolic alkalosis, which is obtained through complex urinary ion excretion mechanisms [2]. Thus, fluid homeostasis depends on the correct relationship between lung and kidney activities because they regulate most of the CO_2_ and hydrogen (H^+^) concentrations in the extracellular volume, whose total solutes consist almost entirely of Na^+^, Cl^−^ and bicarbonate ions (HCO^3−^).

In hypoxic and hypercapnic COPD patients, fluid homeostasis is disturbed, with avid retention of sodium and water [3]. The increase in sodium retention by the kidneys during COPD, and the consequent edema, may be explained in part by right heart failure (cor pulmonale) and by other pathophysiological mechanisms involving renal and hormonal abnormalities [3], [4].

In hypercapnic COPD exacerbations, the sudden decrease in ventilation causes an acute respiratory acidosis or deteriorates a pre-existing chronic respiratory acidosis. Due to the high prevalence of comorbidities [5] and the associated multidrug therapies in these patients, mixed acid-base and hydro-electrolyte disorders are becoming increasingly common, particularly in the critically ill and elderly populations.

This study had the following aims: to evaluate mixed acid-base, hydro-electrolyte and lactate disorders in patients with hypercapnic COPD exacerbation; to determine the relationship among these disorders, a poor response to pharmacological treatment and the requirement for noninvasive ventilation (NIV); and to analyze the link between these disorders and the duration of NIV in the treatment of hypercapnic respiratory failure.

## Methods

### Ethics Statement

The institutional review board for human studies (Fondazione Eleonora Lorillard Spencer Cenci Ethics Committee) for human studies approved the protocol, and written consent was obtained from the subjects or their surrogates.

### Study design

We investigated patients consecutively hospitalized in our respiratory ward (Respiratory Diseases Unit, Policlinico Umberto I, Rome) for COPD exacerbation and hypercapnic respiratory failure. Between January and April 2010, sixty-seven patients were consecutively hospitalized for COPD exacerbation and hypercapnic respiratory failure. COPD and COPD exacerbation were defined according to the Global Initiative for Chronic Obstructive Lung Disease (GOLD) guidelines [6]. Hypercapnia was defined by a PaCO_2_≥45 mmHg on arterial blood gas (ABG) analysis [6].

Patients were excluded from this descriptive study if they had concomitant pneumonia, acute lung injury (ALI) or acute respiratory distress syndrome (ARDS), and contraindications to noninvasive ventilation (NIV) [7]. Patients enrolled were not necessarily at the first COPD exacerbation treated with NIV, but those being on chronic NIV treatment at home were excluded. Comorbidities were identified on the basis of clinical records, concomitant therapy, and/or investigations carried out at hospital admission.

Baseline demographic characteristics and clinical parameters, routine blood chemistry and ABG were assessed at admission.

The level of consciousness was assessed using the Glasgow Coma Scale [8]. The severity of the clinical condition was quantified by the Acute Physiology and Chronic Health Evaluation II score [9].

Patients were classified into pure respiratory acidosis or mixed defect (mixed respiratory acidosis–metabolic alkalosis and mixed respiratory-metabolic acidosis) categories [10]. Hypernatremia and hyponatremia were classified as hypovolemic, euvolemic and hypervolemic [11], [12], according to the clinical assessment, total body water (TBW), relative body water deficit, serum urea to creatinine ratio, serum sodium (sNa^+^), urinary sodium (uNa^+^), plasma osmolality (Posm) and urine specific gravity (SG). TBW was expressed as a percentage of body weight (body weight * correction factor, where the correction factor was 0.6 for men, 0.5 for women and elderly men and 0.45 for elderly women). Body water deficit was calculated using the following equation:
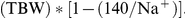
Plasma osmolality was calculated using the following equation:
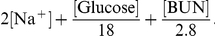
Because the patients' clinical conditions did not allow us to conduct reliable pulmonary function tests, the data (FVC and FEV_1_) considered for the analysis were those measured subsequently at a stable clinical condition (or rather when vital and gas-exchange parameters improved and stabilized and patients' dyspnea was not severe enough to not allow respiratory manouvres required by lung function testing).

Initially, all patients received controlled oxygen therapy with a Venturi mask, in order to maintain an arterial oxygen saturation (SaO_2_) at least ≥90%, and conventional treatments, including nebulized bronchodilators, intravenous corticosteroids, antibiotics (if necessary) and other drugs, depending on the concomitant comorbidities. NIV was initiated when controlled oxygen therapy and convientional medical treatments failed to improve the clinical conditions, or rather when respiratory distress with accessory muscle use or abdominal paradox and tachypnea (respiratory rate >24/min) were associated with persistent arterial impairment (pH<7.35, PaCO_2_>45 mmHg, and PaO_2_/FiO_2_<200) despite optimal pharmacological treatment and oxygen therapy [7], [13]. NIV was performed via an oronasal mask using a pressure support turbine ventilator (Polar 2 Hackermann & Bild Ltd., Crespellano, Bologna, Italy) with a bi-tube connection. The pressure support, PEEP and flow-by trigger values were adjusted to obtain a tidal volume of 6–8 ml/kg and to gain the best oxygenation and reduce the respiratory rate. They were modified based on the blood gas data. Heart and respiratory rates, pH, PaO_2_ and PaCO_2_ and blood lactate were checked on the 1^st^, 2^nd^, 6^th^ and 24^th^ hours after starting NIV.

NIV was withdrawn when the clinical condition improved and stabilized, as defined by the following objective parameters: respiratory rate <24/min, heart rate <110/min, pH>7.35 and SaO_2_>90% on FiO_2_<40% [14]. Patients were a priori considered for invasive mechanical ventilation if the following conditions were present: a worsening of arterial pH and carbon dioxide tension despite correct NIV administration, an onset of coma, cardiovascular instability or poor compliance with the NIV device [15].

### Statistical analysis

All quantitative variables are summarized as mean and standard deviation (SD), and the qualitative variables are summarized as frequency and percentage. The results are reported separately for each of the two groups (NIV use and no NIV use). The statistical analysis was conducted by parametric or non-parametric test, according to the distribution of variables as assessed by the Shapiro-Wilk test. Student's t-test for unpaired data was used to compare the quantitative variables between the two groups (NIV use and no NIV use), while the chi-squared test was used for qualitative variables. The unadjusted odds ratio (OR) for NIV use vs. no NIV use and its associated 95% confidence interval (95% CI) were calculated for each variable by a logistic regression analysis. One-way ANOVA for repeated measures was used with the clinical parameters, ABG results and lactate values to evaluate the effects of NIV use over 24 h. Contrast analysis, a priori specified, was used for comparisons versus previous time points.

The differences in NIV duration among groups of patients identified according to the ABG analysis values (respiratory acidosis, mixed respiratory acidosis–metabolic alkalosis and mixed respiratory-metabolic acidosis) were evaluated using the Kruskal-Wallis H test. The Mann-Whitney U test with Bonferroni correction was applied to evaluate the differences of various parameters at each time point between respiratory acidosis patients and mixed respiratory acidosis–metabolic alkalosis patients. Linear regression analysis was applied to evaluate the relationships between NIV duration and various parameters.

Statistical analysis was performed using SPSS Advanced Statistical 10.0 software (SPSS Inc., Chicago, Illinois, USA).

## Results

Between January and April 2010, 75 patients were consecutively hospitalized for COPD exacerbation and hypercapnic respiratory failure, but 67 were enrolled in the study population (5 patients were excluded for being on chronic NIV treatment at home and the other 3 for the presence of concomitant pneumonia).

**Figure 1:**
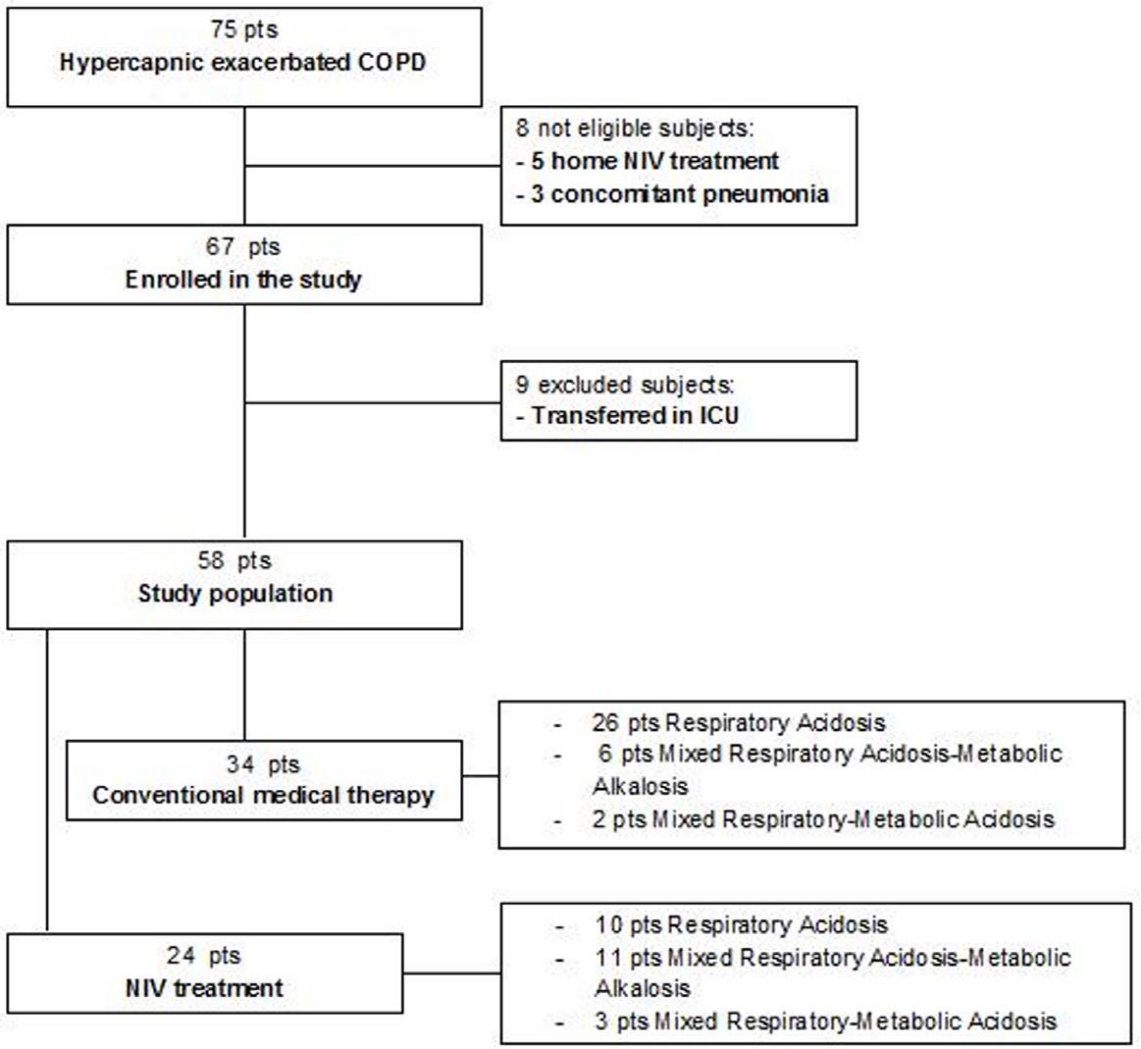
Flow chart of the consecutive COPD patients with acute hypercapnic exhacerbation enrolled in the study.

We considered 58 of the 67 patients initially enrolled, as 9 were excluded because they were transferred to the intensive care unit for pH<7.1, hypercapnic coma or hemodynamic instability (Figure 1).

The enrolled COPD patients had multiple comorbidities, such as hypertension (27 patients, 46.5%); ischemic heart disease (11, 18.9%); degenerative, hypertensive and valvular heart disease (25 patients, 43.1%); atrial fibrillation and other arrhythmias (18, 31%); cor pulmonale (13, 22.4%); peripheral artery disease (9, 15.5%); diabetes mellitus (17, 29.3%); chronic renal failure (20, 34.5%); and obstructive sleep apnea syndrome (4, 6.9%). Therefore they were on multidrugs treatments including diuretics, ACE inhibitors, angiotensin receptor antagonists, digoxin, anti-arrhythmic drugs (such as propafenone, amiodarone, selective beta-blockers and verapamil), oral hypoglycemic agents, insulin, allopurinol, aspirin, statins and inhaled bronchodilators.

NIV was initiated in 24 patients and succeeded in correcting respiratory acidosis in all of them. The mean duration of NIV was 42.4±10.5 hours, and the mean IPAP employed was 16±4 cmH_2_O. Supplementary oxygen was administered during ventilation, which was continuous until clinical conditions were stable with pauses for administration of conventional medications, feeding and general care. None of the patients required the interruption of the ventilatory assistance for discomfort, or refused the treatment. The only complications recorded were 1 case of nasal cutaneous sores and 2 cases of gastric distension, neither of which required interrupting the ventilatory assistance.

Patient demographics and clinical characteristics are shown in table 1. Clinical and metabolic parameters, ABG analysis, electrolyte values, lactate, and urinalysis with electrolytes are shown in table 2.

**Table 1:** Patients demographics and clinical characteristics.

Variables	Total (n = 58)	NIV use (n = 24)	No NIV use (n = 34)	p-value^a^
**Gender:** Male (n, %)	43 (74.2)	18 (75)	25 (73.5)	0.028^b^
**Gender:** Female (n, %)	15 (25.8)	6 (25)	9 (26.5)	
**Age (years)**	79.3±5.1	79.8±5.4	78.8±4.9	0.511
**BMI (Kg/m^2^)**	27.0±2.6	27.3±3.3	26.7±2.1	0.356
**SBP (mmHg)**	138.8±16.9	135.8±17.7	140.9±16.2	0.256
**DBP (mmHg)**	76.7±8.2	74.6±9.8	78.2±6.7	0.097
**FEV_1_ (L)**	2.3±0.7	2.4±0.8	2.3±0.7	0.773
**FEV_1_ (%)**	57.9±9.9	58.6±9.6	57.4±10.2	0.654
**GCS**	12.4±1.6	11.8±1.8	12.8±1.3	0.022
**APACHE II score**	17.7±6.2	18.4±7.5	17.2±5.1	0.477

Data expressed as Mean ± Standard Deviation.

a Student t-test for unpaired data;

b Chi-Squared Test. NIV = non invasive ventilation; BMI = Body Mass Index; SBP = Systolic Blood Pressure; DBP = Diastolic Blood Pressure; FEV_1_ = Forced Expiratory Volume; GCS = Glasgow Coma Scale; APACHE II score = Acute physiology and chronic health evaluation II score.

**Table 2:** Patients main clinical and metabolic parameters, ABG analysis, lactate, serum electrolytes and urinalysis.

Variables	Total (n = 58)	NIV use (n = 24)	No NIV use (n = 34)	p-value^a^
**Glycemia (mg/dl)**	139.5±80.5	150.3±91.2	131.9±72.4	0.396
**Serum albumin (g/L)**	3.9±0.5	4.0±0.5	3.9±0.4	0.339
**Serum uric acid (mg/dl)**	5.7±2.1	5.9±2.5	5.6±1.9	0.651
**Serum creatinine (mg/dl)**	1.3±0.7	1.4±0.7	1.3±0.6	0.798
**Heart rate (beats/minute)**	116.7±11.7	119.7±12.4	114.6±10.9	0.108
**Respiratory rate (breaths/minute)**	29.7±4.0	32.2±3.0	27.9±3.6	<0.001
**PaO_2_ (mmHg)**	59.7±6.7	54.5±4.6	63.4±5.4	<0.001
**PaO_2_/FiO_2_**	178.6±38.5	160.5±38.2	191.5±33.6	0.002
**P(A-a)O_2_**	20.2±6.1	18.2±5.6	21.6±6.1	0.037
**pH**	7.31±0.06	7.22±0.04	7.34±0.04	<0.001
**PaCO_2_ (mmHg)**	68.6±8.3	75.6±5.5	63.7±6.2	<0.001
**Lactate (mmol/L)**	1.7±0.2	3.1±0.6	0.7±0.2	<0.001
**Serum HCO_3_^−^ (mmol/L)**	33.6±7.8	35.6±10.5	32.1±5.0	0.089
**sNa^+^ (mmol/L)**	133.4±9.8	129.4±10.9	136.2±8.0	0.008
**sK^+^ (mmol/L)**	4.0±0.7	3.9±0.9	4.0±0.5	0.683
**sCl^−^ (mmol/L)**	98.1±6.1	95.0±6.8	100.2±4.5	0.001
**sCa^2+^ (mmol/L)**	1.21±0.2	1.19±0.1	1.23±0.1	0.645
**sMg^2+^ (mmol/L)**	1±0.3	1±0.2	0.9±0.3	0.592
**Plasma osmolality (Posm)**	304.9±17.9	309.7±21.4	301.4±14.5	0.084
**uNa^+^ (mmol/L)**	141.5±87.2	123.1±82.2	154.5±89.4	0.178
**uK^+^ (mmol/L)**	90.3±28.6	101.3±32.0	85.2±30.0	0.055
**uCl^−^ (mmol/L)**	168.2±62.5	176.8±69.8	162.1±57.0	0.384
**Urine specific gravity**	1025.7±9.1	1025.6±9.1	1025.7±9.2	0.980

Data expressed as Mean ± Standard Deviation.

a Student t-test for unpaired data. ABG = arterial blood gases; NIV = non invasive ventilation; FiO_2_ = Fraction of inspired O_2_; P(A-a)O_2_ = alveolar-arterial PO_2_ gradient; sNa^+^ = serum sodium; sK^+^ = serum potassium; sCl^−^ = serum chloride; sCa^2+^ = serum calcium; sMg^2+^ = serum magnesium; uNa^+^ = urine sodium; uK^+^ = urine potassium; uCl^−^ = urine chloride.

**Figure 2:**
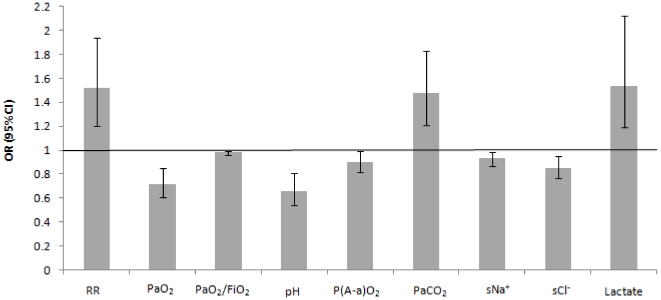
Unadjusted odds ratios (OR) and 95% confidence intervals for NIV use with respect to clinical parameters, ABG results and lactate and electrolyte values that were statistically significant. NIV = noninvasive ventilation; ABG = arterial blood gases; RR = respiratory rate; P(A-a)O_2_ = alveolar–arterial PO_2_ gradient; sNa^+^ = serum sodium; sCl^−^ = serum chloride.

Compared to patients undergoing standard therapy, subjects requiring NIV had a higher respiratory rate; lower PaO_2_, PaO_2_/FiO_2_, alveolar–arterial PaO_2_ gradient, Na^+^, Cl^−^, pH and serum lactate; and higher PaCO_2_ and blood lactate (Table 2, figure 2).

The changes in clinical parameters, ABG results and lactate values during the first 24 hours of ventilation are shown in table 3.

**Table 3:** Clinical parameters, ABG analysis and lactate values at different time during NIV.

Variables	1 h	2 h	6 h	24 h	p-value^a^
**Heart rate (beats/min)**	113.4±14.7	111.3±14.8**	106.9±16.8**	100.3±17.8**	<0.001
**Respiratory rate (breaths/min)**	29.6±4.9	28.5±4.7**	26.0±5.0**	22.6±4.4**	<0.001
**pH**	7.26±0.07	7.38±0.07**	7.31±0.07**	7.35±0.06**	<0.001
**PaO_2_ (mmHg)**	59.3±8.9	61.8±9.3*	64.3±10.0**	66.5±10.1*	<0.001
**PaCO_2_ (mmHg)**	68.0±12.2	66.5±12.2*	62.7±12.5**	59.5±11.7**	<0.001
**Lactate (mmol/L)**	2.9±0.6	2.6±0.7**	2.1±0.8**	1.4±0.8**	<0.001

Data expressed as Mean ± Standard Deviation.

a ANOVA test for repeated measures;

** p<0.001;

* p<0.01 contrast analysis vs previous time point. ABG = arterial blood gases; NIV = non invasive ventilation.

**Figure 3:**
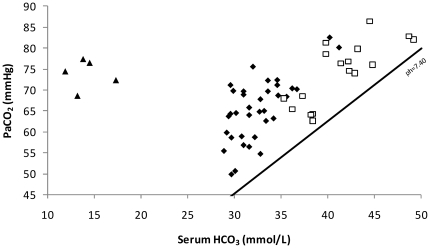
Acid-base balances in patients with hypercapnic COPD exacerbation. *Black lozenge*: Respiratory acidosis. *White square*: Mixed respiratory acidosis–metabolic alkalosis; *Black triangle*: Mixed respiratory-metabolic acidosis.

Using the ABG data and assessing the SID, we identified 36 patients with pure respiratory acidosis (anion gap 11.4±2.3 mmol/L; SID 40.7±1.2 mmol/L), 17 patients with mixed respiratory acidosis–metabolic alkalosis (anion gap 9.6±1.5 mmol/L; SID 49.1±2.0 mmol/L) and other 5 with mixed respiratory-metabolic acidosis (anion gap 25.9±3.6 mmol/L; SID 29.2±1.8 mmol/L) (Figure 3).

Patients with mixed respiratory acidosis–metabolic alkalosis were more likely to use NIV than those with the pure respiratory acidosis, and their NIV duration was longer (Table 4). NIV was used for 3 of the patients with mixed respiratory-metabolic acidosis, and these patients had the longest ventilation times (Table 4).

**Table 4:** Duration of NIV in groups of patients according to ABG analysis values.

Patients	Overall	NIV use (n, %)^a^	Hours of NIV (Mean ± SD)^b^
**Overall**	58	24, 41.4	42.4±10.5
**Respiratory acidosis**	36	10, 27.8	36.2±8.9
**Mixed respiratory acidosis – metabolic alkalosis**	17	11, 64.7	45.1±9.8
**Mixed respiratory - metabolic acidosis**	5	3, 60.0	53.3±4.1

NIV = non invasive ventilation; ABG = arterial blood gases.

a p = 0.026, Chi-Squared test vs no NIV use.

b p = 0.016, Kruskal-Wallis H test.

NIV duration was significantly associated with higher blood lactate, lower pH and lower serum Na^+^ and Cl^−^ concentrations, as shown by their regression coefficients (b = 17.038 and p<0.001 for lactate, b = −111.975 and p = 0.016 for pH, b = −0.478 and p = 0.014 for Na^+^, and b = −0.660 and p = 0.038 for Cl^−^).

**Figure 4:**
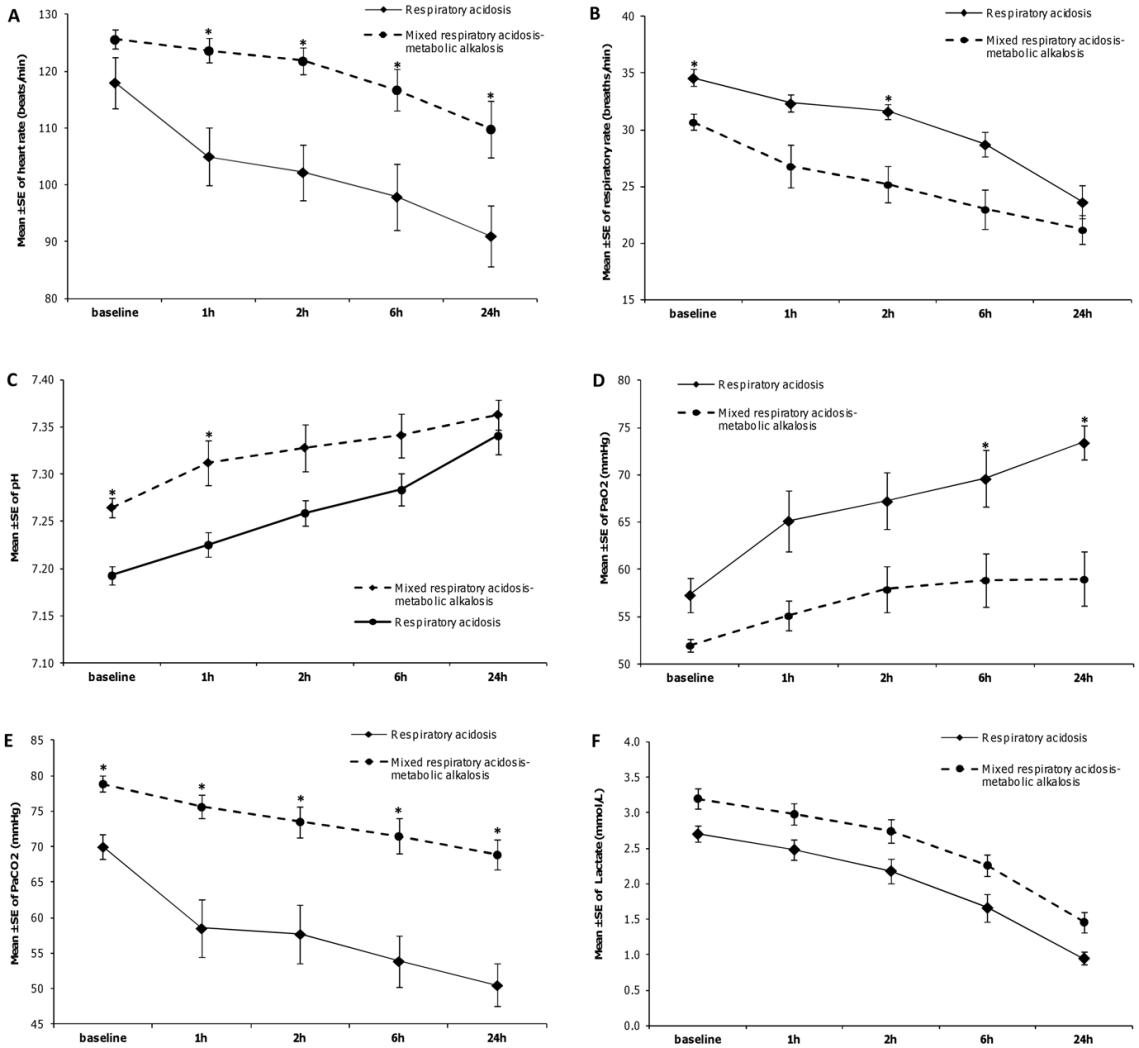
Trends over time in heart rate (panel A), respiratory rate (panel B), pH (panel C), PaO_2_ (panel D), PaCO_2_ (panel E) and blood lactate (panel F) during NIV. * p<0.0125, Mann-Whitney U test between groups with Bonferroni correction. NIV = noninvasive ventilation.

In respiratory acidosis and mixed respiratory acidosis–metabolic alkalosis NIV patients, heart rate, respiratory rate, pH, PaO_2_, PaCO_2_ and blood lactate improved in the first 6 hours of NIV (Figure 4), while significant improvements in the same parameters were observed between the 6^th^ and the 24^th^ hour of ventilation in the mixed respiratory-metabolic acidosis patients (data not shown).

We also evaluated hydro-electrolyte balance and identified 11 respiratory acidosis patients who had normochloremic hypervolemic hyponatremia due to chronic renal failure (n = 2), cirrhosis (n = 1), hypothyroidism (n = 1), or chronic congestive heart failure (n = 7).

Of the patients with mixed respiratory acidosis–metabolic alkalosis, 10 had hypovolemic hyponatremia, hypochloremia and hypokalemia due to thiazide or loop diuretic use associated with diarrhea and vomiting during a gastroenteritis episode (n = 2), profuse sweating from febrile influenza syndrome (n = 4) or (probably) poor dietary fluid intake (n = 4). Euvolemic hypochloremia due to thiazide or loop diuretic use not associated with extrarenal hydro-electrolyte loss was detected in 7 patients with mixed respiratory acidosis–metabolic alkalosis.

In mixed respiratory-metabolic acidosis patients, the metabolic acidosis was due to an exacerbation of chronic renal failure (n = 1), rhabdomyolysis (n = 1), ketoacidosis in decompensated insulin-dependent diabetes mellitus (n = 2) or metformin-related lactic acidosis (n = 1 obese diabetic patient).

## Discussion

Hypercapnic respiratory failure during acute COPD exacerbation is an alarming event that requires careful management of the resulting respiratory acidosis. The final outcome depends on various factors, such as the patient's overall health status and concomitant comorbidities, the baseline lung function, and the disease severity as judged by the need for assisted ventilation and the degree of acidosis [16]. Our observations provide evidence that mixed acid-base and lactate disorders in patients with hypercapnic respiratory failure due to COPD exacerbation lead to the need for and longer duration of NIV. More data should be provided to evaluate this association with combined mixed acid-base and hydroelectrolyte disorders.

### Mixed respiratory acidosis–metabolic alkalosis

We observed that metabolic alkalosis with hyponatremia and/or hypochloremia aggravated the respiratory acidosis due to the COPD exacerbation. Mixed respiratory acidosis–metabolic alkalosis patients were more likely to use NIV and were subjected to longer periods of ventilation compared to those with pure respiratory acidosis. The requirement for and duration of NIV was associated with low serum sodium and chloride, common findings in diuretic-induced metabolic alkalosis. The clinical parameters and ABG analysis indicated more severe ventilatory impairment in the patients with mixed respiratory acidosis–metabolic alkalosis than in those with pure respiratory acidosis, with the exception of those with an elevated pH due to a simultaneous alkalinizing processes.

In patients with hypercapnic respiratory failure due to COPD exacerbation, the presence of a sufficient metabolic compensation and adequate renal function significantly decreases mortality [17]. In our study, the bicarbonate increase overcame the expected renal compensatory response, reflecting a mixed acid-base disorder with metabolic alkalosis due to various causes in patients with multiple comorbidities and undergoing multidrug treatment. The use of diuretics for cardiovascular comorbidities was the main cause of metabolic alkalosis with hyponatremia and/or hypochloremia.

Metabolic alkalosis causes a direct depression of the respiratory drive, leads to diminished chemoreceptor stimulation and consequently reduces alveolar ventilation to increase PaCO_2_ and lower pH toward normal levels [18], [19], [20].

Metabolic alkalosis is usually associated with hypochloremia, which has a relevant inhibitory effect on the ventilatory response to hypercapnia. A reduction of serum chloride is associated with a reduced chloride concentration in the cerebrospinal fluid [21]. Because the cerebrospinal fluid does not contain significant weak acids, a reduction of its chloride level will result in a bicarbonate increase to maintain electroneutrality, which raises the central pH and leads to hypoventilation.

In addition, metabolic alkalosis has been associated with increased airway resistance [22]. In our study, it probably contributed to the increased respiratory impairment in the mixed respiratory acidosis–metabolic alkalosis patients when compared to the subjects with pure respiratory acidosis.

In a study investigating the effects of metabolic acid-base changes on central ventilatory chemoregulation in normocapnic and hypercapnic COPD patients, the ventilatory responsiveness to carbon dioxide was not significantly altered by the induced metabolic state [23]. These results may seem inconsistent with our observations, but we believe that they are not comparable for several reasons. In the van de Ven et al. study [23], chronic metabolic acidosis and alkalosis were experimentally induced by the oral administration of acetazolamide and furosemide, respectively, and were not caused by various comorbidities from their multidrug treatments. The authors evaluated the responses of inspired ventilation (V_I_) and mouth occlusion pressure (P_0.1_) to changes in PaCO_2_, while we investigated the possible deleterious effects of metabolic alkalosis on the ventilatory response to hypercapnia by assessing the requirement and duration of NIV in a real clinical scenario.

The effect of metabolic alkalosis on the clinical outcome of COPD patients was first reported in a study that showed an improvement in gas exchange and clinical symptoms after correcting the coexistent metabolic alkalosis [24].

Discontinuing furosemide, which is prescribed for peripheral edema in patients with stable COPD, increases the minute ventilation and lowers the PaCO_2_ by correcting metabolic alkalosis [25]. Hypoventilation in response to metabolic alkalosis has serious implications in patients with high PaCO_2_ and low PaO_2_. COPD patients with a tendency toward hypercapnia require extra attention because hypercapnia is believed to be an ominous sign for morbidity and mortality [26], [27]. The increased PaCO_2_ caused by furosemide may lead to fluid retention, which may counteract the diuretic effects of furosemide. This is of clinical relevance, especially considering that loop diuretics are prescribed to a substantial number of COPD patients. Thus, other types of diuretics should be considered when diuretic therapy is indicated in hypercapnic COPD patients. Spironolactone, which causes no acid/base shifts, or acetazolamide, which causes a metabolic acidosis, may be better options. Because loop diuretics are often necessary in COPD patients with cor pulmonale or cardiac comorbidities, acetazolamide could be added to counteract the metabolic alkalosis [28].

### Lactate disorders

Lactate clearance, as a surrogate for the magnitude and duration of global tissue hypoxia, is used for diagnostic, therapeutic and prognostic purposes. The normal reference values for lactate are traditionally considered 1±0.5 mmol/l in normal patients and <2 mmol/l in critically ill patients [29].

Increased lactate production indicates increased anaerobic metabolism associated with tissue hypoxia; lactate is one of the intermediate products that increase as a consequence of metabolism derangement during hypoxia [29].

Lactate clearance is a useful prognostic indicator. It reveals improvements in systemic tissue hypoxia and is associated with a decreased mortality rate. Patients with greater early lactate clearance after 6 hours of intensive care treatment have better outcomes than do subjects with lower lactate clearance [30].

In addition, early lactate clearance is significantly associated with decreased levels of inflammatory biomarkers, improvements in organ dysfunction and improved outcomes in severe sepsis and septic shock [31]. In addition, persistently elevated lactate is associated with a higher mortality rate [32], [33].

In patients with respiratory failure, the early lactate clearance could be useful to assess the metabolic response to therapy and to identify individuals who are able to reverse the metabolic derangement. Lactate clearance deserves the same diagnostic relevance as other noninvasive markers of delivery/consumption/demand mismatches. While tissue pH, O_2_ saturation and PCO_2_ can offer a precise “local” picture of cellular oxygen disturbance, lactate does not. Nevertheless, systematically checking lactate clearance could be used to tailor the therapy in many cases of respiratory insufficiency [34], [35].

The greatest amounts of lactate are produced by the skin, erythrocytes, skeletal muscles, and leukocytes [36]. Blood lactate concentration may only qualitatively reflect the metabolic phenomena occurring in the tissues. Moreover, lactate production is actually a consequence (and not the cause) of cellular acidosis. Because increased lactate production coincides with acidosis, lactate measurement is an excellent “indirect” marker of the metabolic condition of a cell.

We considered blood lactate as a diagnostic marker of tissue hypoxia and respiratory muscle fatigue (lactate is a valuable energy source within the working muscle), an indirect indicator of hypercapnic COPD exacerbation severity and, as a consequence, an indirect indicator of its response to treatment.

In our study, lactate improved over time once NIV was started. Patients with higher blood lactate were more likely to require NIV and needed longer ventilation times. The effects of hyperlactatemia on pH were attenuated in patients with concomitant hypochloremic metabolic alkalosis. Therefore, acid-base analysis should not only be based on traditional parameters, such as pH, HCO_3_^−^ and the anion gap, but should rely on a more comprehensive evaluation [37]. According to Stewart [1], lactate production modifies the SID and thus influences one of the determinants of H^+^ concentration. While there are tissues whose deranged metabolic pathways need lactate as an intermediate metabolite to survive, the production of lactate itself depends on pH [37]. Moreover, the pH controls the rate of lactate uptake from blood by hypoxic skeletal muscles [38].

### Mixed respiratory-metabolic acidosis

In this study we identified a few patients with mixed respiratory-metabolic acidosis who required long NIV periods. Metabolic acidosis stimulates hyperventilation, but these patients had impaired ventilation, probably as a result of muscle wasting due to the metabolic academia [39]. Hospitalized patients with acute COPD exacerbations associated with mixed respiratory-metabolic acidosis represent more severe cases with a higher mortality rate than those with the respiratory component of acidemia alone [40].

### Preliminary results and further research

In this descriptive study, we did not analyze the subgroups derived from the combinations of mixed acid-base disorders and hydro-electrolyte disturbances because the number of patients was not sufficient to guarantee a significant statistical power for this purpose

Probably the descriptive design of the study does not allow us to obtain definitive conclusions, but we strongly believe to have investigate a field not yet explored, eventually stimulating further prospective multicentric studies in order to completely clarify this topic, which recurs frequently in clinical practice.

Our preliminary results suggest, in fact, that in the clinical respiratory care researchers should address the following questions: a) whether lactate clearance is useful during acute respiratory failure to identify patients at high risk of negative outcomes and, potentially, to increase the intensity of the therapeutic approach; b) whether lactate clearance is predictive of positive outcome and could confirm to physicians that the current therapeutic approach is appropriate; and c) how both the metabolic components of mixed acid-base disorders and the hydroelectrolyte balance alterations may worsen the hypercapnic respiratory failure caused by the COPD exacerbation and may affect its resolution through standard medical therapy, either alone or combined with NIV.
